# Chlorobarium

**Published:** 1897-02

**Authors:** 


CHLOROBARIUM. After some fifty cases of colic had been treated in Dieckerhoff's clinic in Berlin with chlorobarium and the reports were published, and after the unusual effect of this drug was reported from many quarters, E. Zechokke was led to make some experiments with the same remedy in the Zurich clinic. According to Dieckerhoff, an effect is obtained in fifteen to forty-five minutes, when the medicine is administered per os, the average dose for horses being ten grains, either in pills or in solution. On the other hand, a much more prompt result is obtained with subcutaneous or intravenous injections. A physiological test must give us the indications for its use in the case of sick animals; therefore, we made the first tests on healthy horses, and for that purpose we choose seven animals in ordinary condition. Intravenous injections were made at first. This operation is easy and without danger. Cut off the hair around the jugular vein, disinfect the spot, cause the vein to swell up a little by compression as when bleeding, and place in a syringe eight or ten grains diagonally up or downward. Chlorobarium, which comes in pure white crystals with a salty taste, dissolves easily in water and is usually administered in a 10 per cent. solution. Like all barium salts, chlorobarium is a powerful poison to man. The horses were numbered and their normal condition noted. They were of average size, somewhat old, but all robust. The injections were made in all the animals at almost the same time, and each animal was watched and guarded by a student for one and one-half hours, until every symptom had disappeared. From the 10 per cent. solution three horses were injected with 10 cubic centimetres, two with 7 cm., one with 5 cm., and one with 15 cm. The first reaction followed in all cases in from three to thirty seconds, in the form of twitchings at the mouth, which continued from fifteen to thirty minutes. The horses did not eject any saliva, however, but twisted and shook their heads in a peculiar manner, giving the impression that the effects were unpleasant to them. The effect on the stomach was visible in thirty to one hundred and fifty seconds. The animals raised their tails and emitted gas. After one and a half to three and a half minutes emissions of solid excrement followed without exertion. Signs of restlessness now presented. The horses moved about uneasily, drew themselves together, and looked around toward their hinder parts. The horse with the 1.5-grain dose began to groan and move violently. In the horses with smaller doses these symptoms were present only to a slight degree. One even began to look around for food after four minutes. Soon the evacuations began to increase, as did the emission of gas. Within the first hour the animals evacuated respectively 5, 7, 12, 13, 14, 14, 26 times. At first the dung was hard; later it became watery. One animal moved and groaned continually; a second lay down; in none was there any sweating or drivelling. One animal urinated with considerable effort. While no increase of temperature could be noted, the pulse increased in most cases four to eight beats—in one case thirty-six beats, although this animal kept relatively quiet. Dieckerhoff's obsevation that chlorobarium affects the heart was confirmed by this. However, this symptom was only transient, as the reaction lasted in some animals only three-quarters of an hour; in others one and one-half hours, after which they all appeared perfectly well. I am of the impression that chlorobarium increases the peristalsis of the stomach more certainly and promptly than any other drug without increasing the secretion of the glands. Since it is not always possible in cases of colic to make an intravenous injection, eight days later subcutaneous injections were made upon six horses, as follows: in two, 5 grains; in two, 10 grains; in one, 15 grains; and in one 20 grains of the 10 per cent. solution. The injections were made behind the shoulder. In from one-half to one minute on an average nervous twitching of the hip occurred, but did not last so long as in the former cases. Very soon, however, all the animals showed marked restlessness and some of them decided symptoms of colic. They scraped and stamped, lay down repeatedly, looked around, and shook their heads. One rolled on the ground, another one was covered with sweat. The evacuations were not notably frequent; they numbered during the period of observation respectively 1, 2, 3, 3, 6, 6 times. The pulse increased from 46 to 56. Respiration and temperature remained normal. Whether the colicky symptoms are really to be attributed to the pain in the stomach alone—the peristalsis being more active than normally—cannot be asserted with certainty. The animals invariably scraped with the limb in which the injection had been made, and tried to gnaw or rub the spot. Although there was no swelling, heat, or pain at these points, yet the injection seems to cause a local excitation. The restlessness lasted one and one-half hours, and in two hours all the horses had regained their normal condition. No general or local disturbance remained at the point of injection. The subcutaneous injections, however, were not satisfactory in their results. Although the evacuations took place, the general symptoms of restlessness, the pains in the stomach, and the local excitation at the point of injection cause such a complexity of symptoms that one is constrained to say that colic is caused instead of being cured. We would, therefore, in agreement with Dieckerhoff, recommend only the intravenous injection. It is not more difficult to perform than the subcutaneous injection, works promptly without any of the disturbing minor symptoms, and does not involve danger if carried out carefully and cleanly.—*Schweizer Archiv für Tierheilkunde*, January and February, 1896.

